# Extranodal Natural Killer/T-Cell Lymphoma, Nasal Type: Basic Science and Clinical Progress

**DOI:** 10.3389/fped.2019.00141

**Published:** 2019-04-16

**Authors:** Yasuaki Harabuchi, Miki Takahara, Kan Kishibe, Toshihiro Nagato, Takumi Kumai

**Affiliations:** ^1^Department of Otolaryngology-Head and Neck Surgery, Asahikawa Medical University, Asahikawa, Japan; ^2^Department of Innovative Head and Neck Cancer Research and Treatment, Asahikawa Medical University, Asahikawa, Japan

**Keywords:** nasal natural killer /T-cell lymphoma, cytokine, chemokine, Epstein-Barr virus, ICAM-1, CCR4, PD-L1, MPVIC-P

## Abstract

Extranodal natural killer (NK)/T-cell lymphoma, nasal type (NNKTL) has very unique epidemiological, etiologic, histologic, and clinical characteristics. It is commonly observed in Eastern Asia, but quite rare in the United States and Europe. The progressive necrotic lesions mainly in the nasal cavity, poor prognosis caused by rapid local progression with distant metastases, and angiocentric and polymorphous lymphoreticular infiltrates are the main clinical and histologic features. Phenotypic and genotypic studies revealed that the lymphoma is originated from either NK- or γδ T-cell, both of which express CD56. In 1990, the authors first reported the presence of Epstein-Barr virus (EBV)-DNA and EBV-oncogenic proteins, and EBV has now been recognized to play an etiological role in NNKTL. *in vitro* studies revealed that a wide variety of cytokines, chemokines, and micro RNAs, which may be produced by EBV-oncogenic proteins in the lymphoma cells, play important roles for tumor progression in NNKTL, and could be therapeutic targets. In addition, it was revealed that the interaction between NNKTL cells and immune cells such as monocytes and macrophages in NNKTL tissues contribute to lymphoma progression. For diagnosis, monitoring the clinical course and predicting prognosis, the measurements of EBV-DNAs and EBV-micro RNAs in sera are very useful. For treatment with early stage, novel concomitant chemoradiotherapy such as DeVIC regimen with local radiotherapy and MPVIC-P regimen using intra-arterial infusion developed with concomitant radiotherapy and the prognosis became noticeably better. However, the prognosis of patients with advanced stage was still poor. Establishment of novel treatments such as the usage of immune checkpoint inhibitor or peptide vaccine with molecular targeting therapy will be necessary. This review addresses recent advances in the molecular understanding of NNKTL to establish novel treatments, in addition to the epidemiologic, clinical, pathological, and EBV features.

## Introduction

Extranodal natural killer (NK)/T-cell lymphoma, nasal type (NNKTL) has very unique epidemiological, etiologic, histologic, and clinical characteristics. The lymphoma is commonly observed in Eastern Asia (1–4) and Latin America (4, 5) but quite rare in United States and Europe (6–8). The progressive necrotic lesion mainly in the nasal cavity is one of the main clinical features of this disease, which is often characterized by a poor prognosis because of rapid local progression and distant metastases (2, 9). The histological feature shows angiocentric and polymorphous lymphoreticular infiltrate, and the disease has been previously called polymorphic reticulosis (10, 11). Phenotypic and genotypic studies revealed that the lymphoma is originated from either NK- or γδ T-cell, both of which express CD56 (2, 8, 12–16). In the late 20th century, the authors first demonstrated the presence of Epstein-Barr virus (EBV) DNA, EBV oncogenic proteins, and the clonotypic EBV genome in NNKTL (1, 2, 16, 17). Based on these findings, EBV has been recognized to play an etiological role in NNKTL (16), and EBV DNA has been applied as a clinical progression/recurrence marker (18). Although NNKTL generally occurs in adult patients, a few cases of pediatric NNKTL are reported (19). In this article, the authors summarize the current understandings of clinical, pathological, biological, and virological characteristics of this lymphoma.

## Historical Backgrounds

McBride (20) first reported, in 1897, the rapid destruction of the nose and face (midline) with progressing necrotic granuloma. The clinical course was generally aggressive and lethal, this disease was initially termed as “rhinitis gangrenosa progressiva” (21) in Europe or “lethal midline granuloma” (22, 23) in the United States. The histologic features show angiocentric and polymorphous lymphoreticular infiltrates with necrotic granuloma. Therefore, the disease had been called many histopathologic terms such as “reticulum cell sarcoma,” “midline malignant reticulosis”, “polymorphic reticulosis” (11), and “malignant histiocytosis” (24). Since the late twentieth century, this disease had been coined as nasal T-cell lymphoma based on the finding that these tumor cells had a T-cell phenotype (9, 25). Subsequently, the expression of NK-cell marker CD56 was also reported. Accordingly, the term of this lymphoma has been determined as nasal NK/T-cell lymphoma (NNKTL) (26).

Etiologically, Harabuchi et al. (1) first found the presence of EBV-DNA and EBV-determined nuclear antigen (EBNA1) in the lymphoma cells from 5 Japanese patients. These EBV-findings were also verified in Western countries (8, 27–29). Accordingly, NNKTL is now classified as one of the EBV-associated malignancies (16).

## Epidemiology

There is a clear geographic deviation in NNKTL prevalence. In Asia and South America, NNKTL consists of 3-10% of non-Hodgkin lymphoma, whereas less than 1% in Western countries (30–33). In Peru, the percentage of NNKTL in non-Hodgkin lymphoma was 8%, respectively (34). Aozasa et al. (35) estimated that the incidence rate of NNKTL is higher in Asia by 10-fold compared to Europe. Because the common race in Asia and South America is mongoloid, the genetic background may play a role in the onset of NNKTL. Although the specific genetic feature of NNKTL patients remains to be elucidated, there is a possibility that a specific type of HLA has the disadvantage to present EBV-associated epitope to T cells. Indeed, HLA-B46 is a risk factor in nasopharyngeal cancer, an EBV-associated malignancy (36).

Another explanation of the skewed distribution of NNKTL could be EBV strain and/or environmental factors. Because the subtype of EBV in NNKTL (Type A/F/C) is similar to healthy donors (37), the high-risk EBV subtype has not been identified in this lymphoma. Nagamine et al. (38) previously demonstrated that the EBV strain in NNKTL has an amino acid mutation in the CD8 T-cell epitope. Furthermore, Nagamine et al. (39) investigated the full length of the LMP1 sequence in NNKTL and found the amino acid changes at codon 126 and 129 in an HLA-A2 restricted CD8 T cell epitope LMP1 125–133 from all patients. Our group (40) previously showed that the frequency of HLA-A^*^0201 was significantly lower in NNKTL than in the healthy population, suggesting that the HLA-A^*^0201-restricted CTL responses may inhibit the development of the lymphoma. It is possible that the mutated EBV allows infected cells to escape from cytolysis by immune cells. The precise characteristics of EBV in NNKTL are described below.

The environmental factors also influence the pathogenesis of NNKTL. Our group (41) previously demonstrated that the exposure to pesticides and chemical solvents could be a significant risk factor in NNKTL by a case-control study. Every type of pesticide, herbicide, insecticide, and fungicide was related to the NNKTL incidence. Moreover, the use of gloves or mask to circumvent the pesticide pollution was effective to reduce the incidence of NNKTL. Kojya et al. (42) also reported the case of familial development of NNKTL who are exposed to the pesticide. Taken together, the genetic background, EBV strain, and environmental factors concordantly affect the geographic distribution of NNKTL.

## Clinical Features

**Figure 1:**
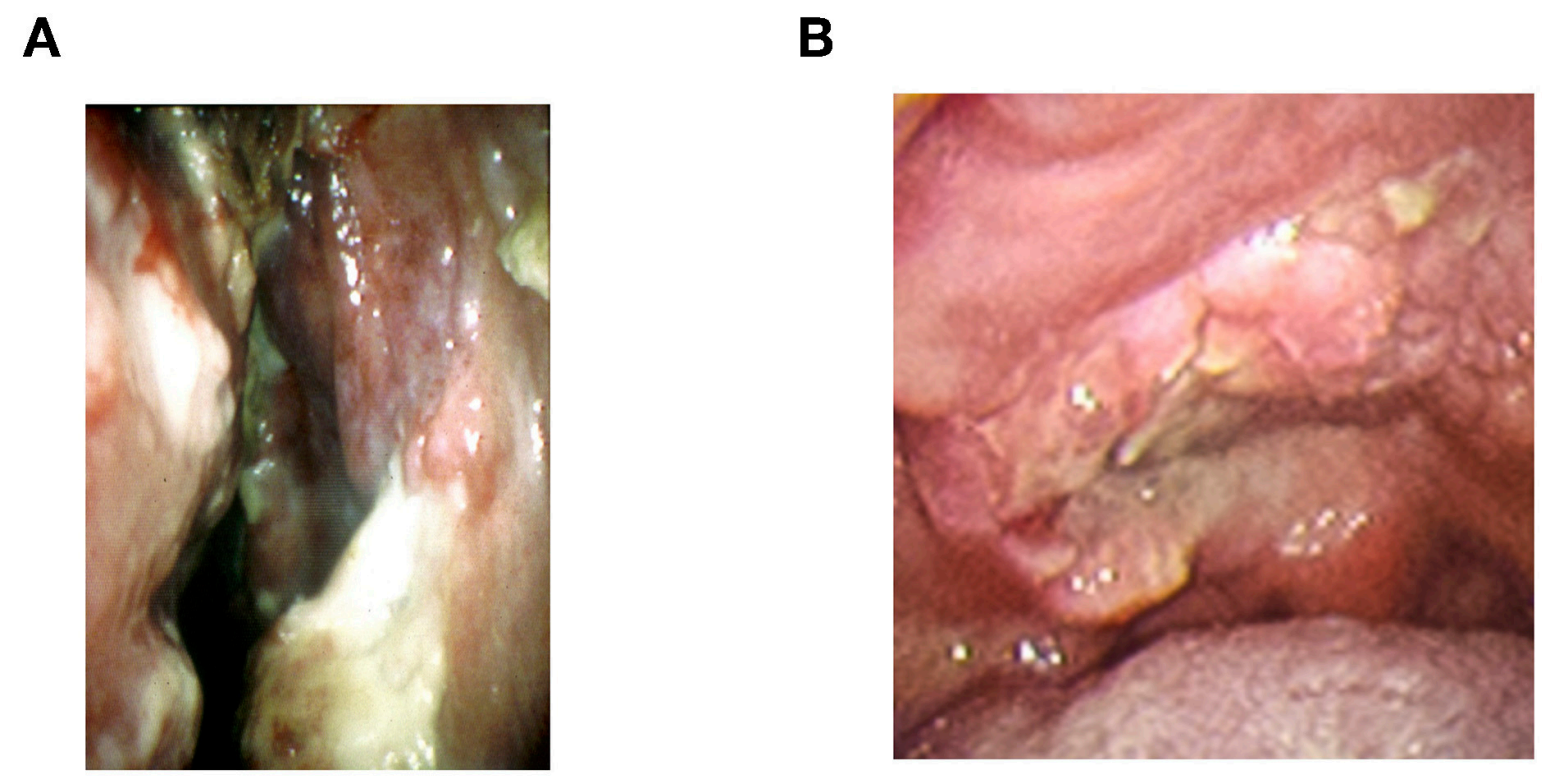
The representative local findings of NNKTL. **(A)** Necrotic granulation in nasal cavity. **(B)** Necrotic ulceration in hard palate.

The lymphoma is initially found as progressive ulceration and necrotic granuloma in the nasal cavity, palate, and nasopharynx (1, 2) (Figure 1). The tumor frequently invades around tissues such as facial skin, paranasal sinus, and orbits, and then develops extensive destruction of midline lesions (1, 20, 23). The most common symptoms at the time of diagnosis are nasal obstruction and bloody rhinorrhea (2, 43). The swelling of cheek or orbit, sore throat, and hoarseness are also major symptoms of NNKTL (2, 43). In addition, systemic symptoms such as prolonged fever and weight loss are commonly seen (2, 43, 44).

The clinical characteristics of NNKTL from different countries are summarized in Table 1 (3, 43–47). The disease developed around 40 to 50 years of age, and there is no significant difference between the sexes. The patients over 60 years old were not common (18–35%). Most patients (69–100%) were diagnosed in early stages, Stage I or II. As mentioned above, the most common symptoms were nasal obstruction (70–80%), bloody rhinorrhea (44–47%) and B symptoms (31–53%). The tumor directly invaded several tissues including nasal cavity, hard plate, facial skin, and pharynx. Lymph nodes and distant tissues such as liver, lung, digestive tracts, and bone marrow were also involved. Twenty to forty percentages of patients had high lactate dehydrogenase (LDH), whereas 38% of patients had high soluble IL-2 receptor in serum. More than 90% of tumors expressed T cell markers such as CD3 or NK marker CD56 on the surface. EBV LMP1 was found in 47% of patients. T-cell receptor (TCR) gene rearrangement was observed in 10–35% of patients. The precise role of EBV, surface markers, or TCR gene rearrangement is described later in this review.

**Table 1:** Clinical characteristics of NNKTL from different countries.

**Reported country**		**Japan**	**Japan**	**China**	**Korea**	**Korea**	**Brazil**
Reported year		2016	2010	2008	2006	2005	2011
Authors		Our institution	Suzuki et al (45)	Wu et al. (43)	Lee et al. (3)	Kim et al. (46)	Gualco et al. (47)
Case number		62	123	115	262	114	122
Age	Range (mean)	20–85(53)	14–89(52)			(47)	9–89(45)
	>60	22(35%)		20(18%)	55(21%)	20(18%)	
Sex	Male/Female	43/19	81/42	78/29	170/92	72/42	85/37
**CLINICAL STAGE**							
	I/II/III/IV	44/13/1/4	55/29/8/31	61/26/8/12		83/31/0/0	23/2/2/4
	I+II(%)	57(92%)	84(68%)	87(76%)	200(76%)	114(100%)	25(81%)
Symptom	Nasal obstruction	49(70%)		84(73%)			97(80%)
	Bloody rhinorrhea	29(47%)		50(44%)			
	B symptom	32(52%)	56(46%)	57(53%)	92(35%)	35(31%)	
Invaded tissues	Nasal cavity	60(97%)	111(90%)	115(100%)		73(64%)	97(80%)
	Hard plate	11(18%)		8(7%)		15(13%)	
	Facial skin	13(21%)	19(15%)				
	Pharynx	13(21%)	28(23%)	27(23%)		21(18%)	
	Lymph nodes	10(16%)	31(25%)	21(18%)			
	Skin	9(15%)					
	Liver	9(15%)	10(8%)		4(2%)		
	Lung	10(16%)	10(8%)		4(2%)		
	Digestive tracts	5(8%)			10(4%)		
	Bone marrow	3(5%)	9(7%)	3(3%)	16(6%)		
	VAHS	3(5%)					
**SEROLOGIC FINDINGS (HIGH CASES/TOTAL CASES)**							
	High LDH	22/61(36%)	52(43%)	28(26%)	96(33%)	34(31%)	
	High sIL-2R	9/24(38%)					
**PATHOLOGIC FINDINGS (POSITIVE CASES/TOTAL CASES)**							
CD3		25/47(53%)	68/86(79%)	105/108(97%)		104(98%)	116/122(95%)
CD43		31/35(89%)	15/17(88%)				
CD45RO		25/35(71%)	44/49(90%)	103/110(94%)		61/62(98%)	
CD20		0/59(0%)	1/14(7%)	0/115(0%)		0/106(0%)	
CD56		61/62(98%)	115/120(96%)	95/105(91%)	262(100%)	94/106(89%)	103/122(84%)
CD16		5/11(45%)	9/40(23%)				
EBER		59/62(95%)	93/94(99%)	106/110(96%)	262(100%)	46/61(75%)	74/74(100%)
LMP1		25/53(47%)					10/122(8%)
TCR rearrangement		12/34(35%)					7/74(10%)

## Pathology

Pathologic characteristics of NNKTL show diffused infiltrates of lymphoma cells, which have a diverse size, pleomorphic large or small cells with mitosis, together with various inflammatory cells such as granulocytes, macrophages and plasma cells, in the necrotic background. The lymphoma had been called as polymorphic reticulosis or angiocentric lymphoma because angiocentric and angio-invasive infiltrates are commonly found (48). The lymphoma cells express T-cell markers such as CD2, cytoplasmic CD3 (CD3ε), and CD45 as well as NK-cell marker CD56. Perforin, Fas ligand, and intercellular adhesion molecule-1 (ICAM-1) are also shown in the NNKTL cells (49).

The lymphoma cell was initially thought to be originated from NK-cells alone by reason that the gene rearrangement of T-cell receptors (TCR) was not found out (50). However, a number of cases with TCR rearrangement reported by Harabuchi et al. (2) and others (51, 52) indicated that some NNKTL are derived from T-cell lineage. This is evidenced by Nagata et al. (13), who succeeded in establishing two NNKTL cell lines from patients, NK-cell lineage without TCR rearrangement and γδ T-cell lineage with γδ TCR rearrangement. Therefore, the current concept of the origin of NNKTL is NK- or γδ T-cells lineage (14, 15) as first proposed by Harabuchi et al. (2).

## EBV Characteristics

We first reported the close association of EBV with NKTCL in 1990 (1), because EBV genomic DNA and EBNA1 were identified in the nuclei of the lymphoma cells. Subsequently, we demonstrated clonotypic EBV genome (2), suggesting that EBV plays a role in the lymphomagenesis. The other studies also confirmed the etiological role of EBV for NNKTL (1, 8, 28, 29). The lymphoma cells express EBNA1 but not the other EBNAs (2, 53). We detected the mRNA of LMP 1 in all patients, but found the protein of LMP 1 in only half of the patients, because of the methylation of LMP coding sequences (2, 17, 44). Therefore, NNKTL is categorized to the type II latency infection of EBV. Moreover, we performed a southern blot analysis of terminal repeats of the EBV genome and found a single fused terminal fragment, indicating that EBV infection may occur at the early stage of lymphomageneses, and EBV infection in these cells is not from contamination (2, 17, 53).

To discover the oncogenic strains of EBV, several efforts have been made. In the sequence analysis of LMP1, the 30-bp deletion in the codon 343–352 of the B95-8 strain was found in the vast majority of the patients (39, 54). Moreover, we found that several amino acid changes in the LMP1 and LMP2 sequence coding major HLA-A2 restricted CTL epitopes (38, 39). These data suggest that the mutation in EBV endows EBV-infected cells with the ability to escape from immune surveillance by CTLs, which may play an important role in lymphomagenesis.

## Gene Mutations

The genetic abnormalities, which may have pathogenic importance, have been reported in NNKTL. The deletion in the chromosome 6q21–25 was frequently found (55–58). The aberrant activation of the JAK/STAT3 pathway, which supports the growth of tumors, has been reported (59). The gene mutations in the cell surface receptor Fas (Apo-1/CD95), which transmits an apoptosis signal, were detected in more than half of the patients (60, 61). Our group found a different frequency of p53, K-ras, and c-kit mutations between NNKTL in Korea and Japan (62). In NNKTL, the p53 mutations were detected in 20–50% of patients (44, 62–65). However, our group showed that mutations of the Ras, c-kit, and β-catenin were not frequent (44, 62, 64). Regarding relation to prognosis, Takahara et al. (44) showed that the p53 missense mutation had a prognostic value predicting poor survival.

## Proliferation and Invasion Factors of NNKTL Cells

Based on the success of establishing two EBV-positive NNKTL cell lines from NK- and γδ T-cell lineage origins (13), the gene or protein expressions of these cell lines have been discovered. Nagato et al. (66) investigated gene expression patterns of these NNKTL cell lines using cDNA arrays, and (66) found that both the IL-9 mRNA and protein were specifically expressed in NNKTL cell lines (66). NNKTL cell lines also expressed IL-9 receptor. Anti-IL-9 neutralizing antibody decreased proliferation of the cells and recombinant IL-9 increased, suggesting that NNKTL cells use IL-9 as a proliferation factor in an autocrine manner. IL-9 was present in clinical specimens and NNKTL patient sera. EBER induces IL-9 expression (67), suggesting that EBV may play a role for IL-9 expression in the lymphoma.

In addition to IL-9, Takahara et al. (68) found that IL-10 was also secreted by NNKTL cells. Exogenous IL-10 increased CD25 (IL-2 receptor) and LMP1 expressions, and then enhanced cell growth of NNKTL. IL-10 treated cells required lower amounts of IL-2 for proliferation. This effect was seen only with the EBV-positive NK-cell lines, in which CD25 and LMP1 were overexpressed, suggesting that IL-10 induces IL-2 receptor expression via enhancement of LMP1 expression, resulting in the proliferation of NNKTL cells.

Chemokines play a huge role in proliferating/recruiting tumor cells and immune cells. Moriai et al. (69) analyzed the expression of chemokines in these NNKTL cell lines using a protein array analysis. We found that the interferon-gamma-inducible protein-10 (IP-10), i.e., CXCL10 was produced in NNKTL cell lines. The amount of IP-10 was significantly larger in NNKTL cell lines than in EBV-negative NK-cell lines. IP-10 was also determined in the biopsy samples and sera from NNKTL patients. The receptor of IP-10, CXCR3, was also expressed in NNKTL cells. *In vitro* studies showed that exogenous IP-10 enhanced invasion of the NNKTL cells, on the other hand, the neutralizing antibodies to IP-10 and CXCR3 inhibited, suggesting that NNKTL cells use IP-10/CXCR3 to invade in an autocrine manner.

Subsequently, Kumai et al. (70) found that NNKTL cells produced chemokine (C-C motif) ligand (CCL) 17 and CCL22. CCL17 and CCL22 were also observed in the NNKTL patients' sera. Moreover, CCR4, which is the receptor for CCL17 and CCL22, was expressed on the NNKTL cell lines and tissues. Anti-CCR4 antibody efficiently induced antibody-dependent cellular cytotoxicity mediated by NK-cells against NNKTL cell lines. Because anti-CCR4 antibody mogamulizumab has shown clinical efficiency in cutaneous T-cell lymphoma (71), this antibody could also be a useful option in NNKTL treatment.

Metalloelastase is a family of extracellular matrix-degrading enzymes. Metalloelastase degrades several substrates such as elastin, laminin, collagen, fibronectin, and casein. Because MMP-9 was expressed in NNKTL samples (16, 72), NNKTL cells might use this enzyme to invade into surrounding tissues.

CD70, a ligand of CD27, is expressed on activated T-cells, B-cells, and lymphoma. Because lymphoma expressed a higher level of CD70 than lymphocytes, anti-CD70 antibodies might be a possible treatment for CD70 positive lymphomas (73). Yoshino et al. (74) found that NNKTL cell lines specifically expressed CD70, but not EBV-positive NK-cell lines without LMP1 did not. Exogenous soluble CD27, which is the ligand for CD70, enhanced cell proliferation of NNKTL cells in a dose-dependent fashion. In the clinical samples, CD70 was expressed on the NNKTL tissues, and soluble CD27 was detected in patients' sera at higher levels. These results suggest that soluble CD27/CD70 signaling, possibly up-regulated by LMP-1 (75), supports lymphoma progression, and anti-CD70 antibody may be a candidate for the NNKTL treatment.

Intercellular adhesion molecule (ICAM)-1, a ligand for LFA-1, attracts macrophage and create precancerous environment (76). Harabuchi et al. (49) have previously shown that ICAM-1 and soluble ICAM-1 (sICAM-1) was expressed in NNKTL cells and in NNKTL patient sera, respectively. To elucidate the functional role of ICAM-1 in NNKTL, Takahara et al. (77) examined the NNKTL proliferation with sICAM-1. As a result, exogenous sICAM-1 enhanced the proliferation of NNKTL cells, whereas LFA-1/ICAM-1 blockade by anti-ICAM-1 antibody, anti-LFA-1 antibody, or LFA-1 inhibitor simvastatin reduced the number of viable NNKTL cells. In the NNKTL tissues, we confirmed that NNKTL cells also expressed LFA-1. Accordingly, the blockade of LFA-1/ICAM-1 by simvastatin may be a potential agent for NNKTL.

Micro RNAs (miR) play an important role in the carcinogenesis of several malignancies by regulating gene expression. Komabayashi et al. (78) performed MiR array and quantitative RT-PCR analyses and then found that miR-15a was downregulated, while the expression of MYB and cyclin D1 was elevated in NNKTL cells. On the other hand, transfected NNKTL cells with miR-15a precursor downregulated MYB and cyclin D1 levels, resulting in blocking G1/S cell cycle transition and cell proliferation. In NNKTL tissues, we found that the reduced miR-15a expression, which correlated with MYB and cyclin D1 expression, was associated with poor prognosis of NNKTL patients. Knockdown of LMP1 significantly increased miR-15a expression in NNKTL cells, suggesting that LMP1 downregulate miR-15a and then induce cell proliferation via MYB and cyclin D1. Therefore, miR-15a may be useful as a target for anti-tumor therapy as well as a prognostic factor for NNKTL patients.

**Figure 2:**
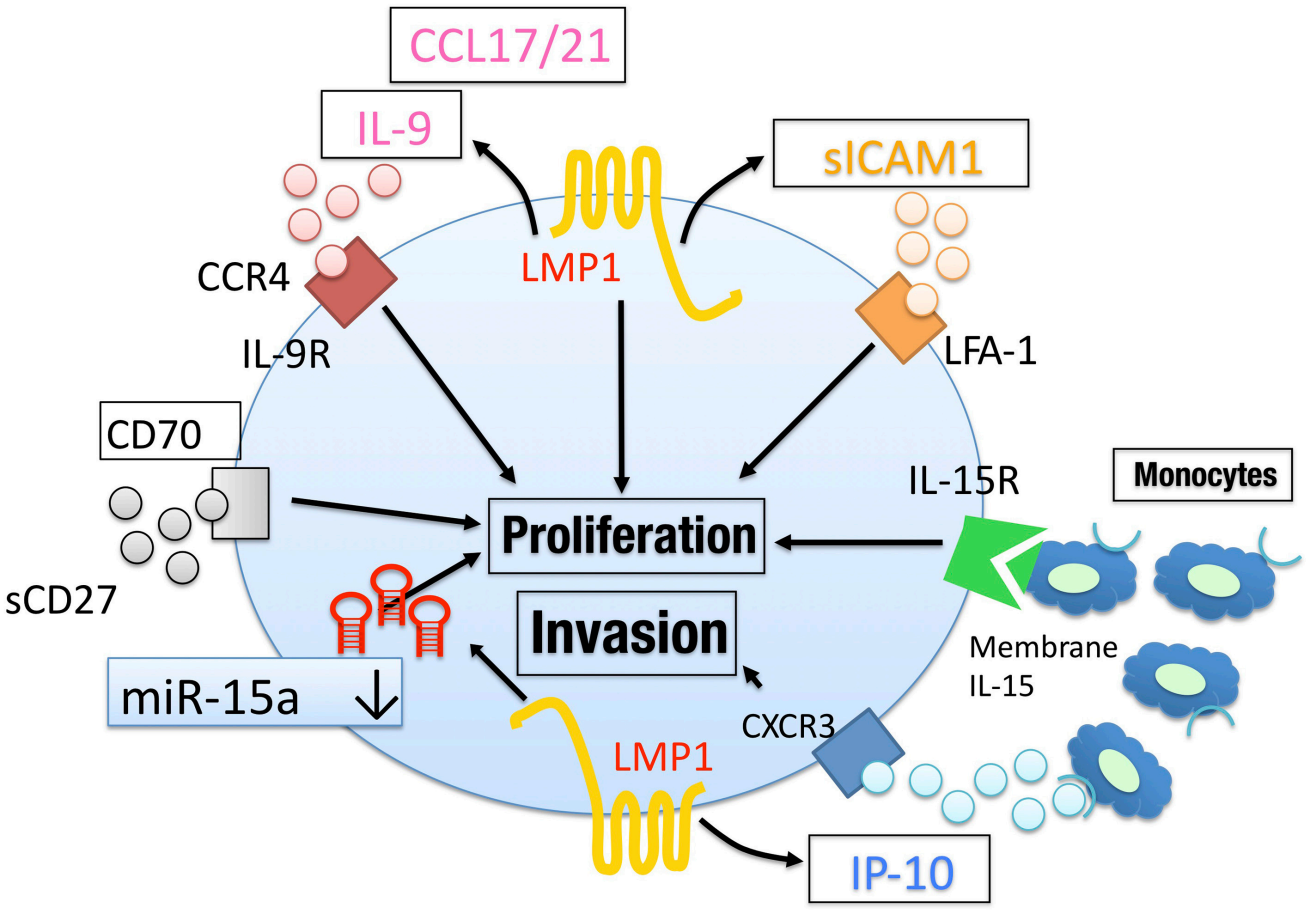
The tumor microenvironment in NNKTL. NNKTL utilize CCL17/21, IL-9, IP-10, and soluble ICAM1 to proliferate/invade in an autocrine manner. These factors may be regulated by EBV LMP1. CD70 activation via soluble CD27 also mediate tumor proliferation. The downregulation of miR-15a mediates tumor progression by regulating surviving. Surrounding immune cells such as monocytes support NNKTL through IL-15 signaling.

Together, these results suggest that cytokines, chemokines, and miR, which may be produced by EBV-oncogenic proteins in the lymphoma cells, play important roles for tumor progression in NNKTL (Figure 2), and could be therapeutic targets in NNKTL patients.

## Interaction Between NNKTL Cells and Immune Cells

Histological features of NNKTL are characterized by diffused infiltrates of lymphoma cells, together with various inflammatory cells such as granulocytes, monocytes, macrophages, and lymphocytes. Accordingly, it is rational to consider that there is an interaction between NNKTL cells and surrounding immune cells. Ishii et al. (79) co-cultured the NNKTL cells with monocytes or granulocytes to examine whether proliferation, survival and LMP1 expression of NNKTL cells are affected by immune cells. Although granulocytes had no effect on proliferation, survival, or LMP1 expression, co-cultured monocytes enhanced proliferation, LMP1 expression and IP-10 production of NNKTL cells. Being not observed when monocytes were placed in a separate chamber, this interaction was mediated in a contact-dependent manner. Because the monocyte-induced proliferation and LMP1 expression of NNKTL cells were inhibited by anti-IL15 antibody, monocytes might support NNKTL cells via membrane-bound IL-15/IL-15 receptor α complex. Because NNKTL cells secrete IP-10 (69), CCL2 and CCL22 (70) that are monocyte-attractant chemokines, a positive feedback loop by the interaction between NNKTL cells and monocytes may contribute to lymphoma progression *in vivo* (79).

Recently, the detrimental effects of negative immune checkpoints have been considered as a druggable target (80). Nagato et al. (81) detected the PD-L1 expression on NNKTL cells and PD-1 expression on the macrophages infiltrated the NNKTL tissues. Soluble PD-L1 was also detected in sera of NNKTL patients at higher levels. Patients with higher soluble PD-L1 in sera showed worse prognosis. Furthermore, Nagato et al. (81) elucidated that NNKTL cell lines expressed and secreted PD-L1 *in vitro*. Because IL-10 converts macrophage to tumor-associated macrophages (82), it is possible that NNKTL cells educate macrophage to be tumor-supportive. The clinical significance of PD-1/PD-L1 blockade is mentioned below.

## Diagnosis

Early diagnosis of NNKTL is essential to treat patients promptly (2, 16). Although pathological examination (detection of tumor cells with CD56 and EBER1) is indispensable, the surrounding necrotic tissue may lead to the difficulty of NNKTL diagnosis.

Circulating cell free EBV DNA levels measured by RT-PCR is previously reported to be useful as a tumor marker of EBV-associated malignancies (83). Nagato et al. (18) measured both Bam HI W DNA and LMP1 DNA levels in sera and showed that measurement of both DNAs is more useful as a predictor for prognosis and as a monitoring marker for the clinical course of NNKTL than measurement of Bam HI W DNA alone. These DNAs were decreased with the treatment and increased at relapse in NNKTL patients. Patients with high pre-treatment EBV DNAs showed an aggressive clinical course. Multivariate analysis revealed that high pre-treatment level of both EBV DNAs has the most value as an independent prognostic factor. Because the detection of serum EBV DNA reflects the residual lymphoma cells, further treatment should be considered to achieve a complete remission in NNKTL patients with detectable serum EBV DNAs even after the initial therapy (18). This is supported by prospective measurement of serum EBV-DNA in NNKTL patients (84).

Epstein-Barr virus encodes viral miRNAs (miRs). Komabayashi et al. (85) investigated whether the circulating EBV-miRs level was useful as biomarkers for NNKTL. As a result, the serum levels of miR-BART2-5p, miR-BART7-3p, miR-BART13-3p, and miR-BART1-5p could distinguish NNKTL patients from normal donors. *In vitro* studies confirmed that these EBV-miRs were secreted from NNKTL cells. In NNKTL patients, these levels significantly decreased after treatment. Moreover, a high circulating miR-BART2-5p level correlated with poor prognosis. Thus, circulating EBV-miRs, particularly miR-BART2-5p, are useful as diagnostic and prognostic biomarkers in NNKTL patients.

As described above, Nagato et al. (81) clearly showed that the level of serum sPD-L1 was elevated and consistent with disease prognosis in NNKTL patients. Collectively, serum EBV DNA, miRs, and sPD-L1 must be useful biomarkers in NNKTL treatment.

## NNKTL Treatment

Because a high recurrence rate was reported in the radiation therapy alone (86), chemoradiotherapy is the main strategy to treat NNKTL, but even in early clinical stages, five-year survival rates had been around 50% (44, 87). To improve the treatment outcome, the phase I/II trial (JCOG0211), which consists of three course of DeVIC chemotherapy (dexamethasone, etoposide, ifosfamide, and carboplatin) concomitant with local radiotherapy (50 Gy) for localized NNKTL, conducted in Japan and then showed a good clinical outcome for NNKTL (88). Ifosfamide and carboplatin were chosen because they are not affected by multidrug resistance genes 1, which is frequently expressed in the NNKTL cells (89). Etoposide was used to prevent virus-associated hemophagocytic syndrome (VAHS) (90). Toxicities of the therapy were comparable to those in a previous trial. With a median follow-up of 32 months, 2-year overall survival was 78% (91).

Other regimens such as SMILE (steroid, methotrexate, ifosfamide, L-asparaginase, and etoposide) showed a promising clinical outcome even in a late stage of NNKTL patients (92). Due to the high rate of progression, asparaginase needs to be combined in the regimen with ifosfamide, methotrexate, etoposide, and prednisolone (93). The similar regimen without ifosfamide but sandwiched with radiotherapy also displayed a favorable result (94). The necessity of methotrexate has been examined in the ongoing clinical trial (NCT00283985). The same regimen without radiotherapy has shown satisfactory results (95). Another regimen of the sandwich protocol was reported, Jiang et al. presented the protocol using L-asparaginase, cisplatin, dexamethasone and etoposide sandwiched with radiotherapy (96). The overall response rate in a phase 2 study of sequential radiation therapy followed by gemcitabine, dexamethasone, and cisplatin was 97.5% in early stage NNKTL (97). Another chemotherapy combining gemcitabine (DDPG: cisplatin, dexamethasone, gemcitabine and pegaspargase) without radiotherapy has also shown promising results (98). Li et al. demonstrated that DDPG chemotherapy showed an improved response rate without severe toxicity like the SMILE regimen (99). GELAD (gemcitabine, etoposide, pegaspargase, and dexamethasone, NCT02733458), GDP (gemcitabine, cisplatin, dexamethasone) with radiotherapy (NCT02276248), P-Gemox (gemcitabine, oxaliplatin, and pegaspargase) have been tested in the clinical trials.

Bone marrow transplant is another approach to treat NNKTL. Despite the expectation, the outcome of autologous or allogenic bone marrow transplant is controversial (100–102). Because improved treatment approaches were needed for localized NNKTL exhibiting elevated pretreatment soluble interleukin-2 receptor (103), it is mandatory to develop novel treatment approaches in NNKTL.

Recently, Takahara et al. (104) have developed a novel arterial infusion chemotherapy from a superficial temporal artery in combination with radiotherapy. The regimen for the arterial infusion consists of methotrexate, peplomycin, etoposide, ifosfamide, carboplatin, and prednisolone (MPVIC-P), which is not influenced by multidrug resistance genes 1 (except for etoposide) as well as a DeVIC regimen. Chemotherapy and concomitant radiotherapy were performed for 3 cycles and over 54Gy, respectively. We administered 12 Japanese patients with stage I-II. During the observation period from 39 to 111 months after the treatment (median: 81 months), all 12 patients achieved complete remission and have remained tumor-free. Common adverse effects were mucositis (83%) and myelosuppression (33%), both of which were manageable. Thus, this MPVIC-P regimen using intra-arterial infusion with concomitant radiotherapy is an effective treatment for early stage NNKTL with adaptable toxicity.

## Establishment of Novel Treatments for NNKTL

### Immune Checkpoint Inhibitor

One of the negative immune checkpoints, the PD-1/PD-L1 pathway, plays an important role in immune evasion of tumor cells through T-cell exhaustion. Because of the expression of PD-L1 on tumor cells (81), PD-1 inhibitor is a promising therapeutic armamentarium in NNKTL. Although the general clinical outcome of NNKTL patients failing chemotherapy is fatal, PD-1 inhibitor has shown remission in these patients (105, 106). The adverse events were tolerable. Several clinical trials with PD-1/PD-L1 blockade are ongoing (NCT03595657, 03107962, 03439501). Thus, PD-1/PD-L1 blockade could be a favorable treatment in chemotherapy-resistant NNKTL patients.

### Peptide Vaccine With Molecular Targeting Therapy

Among immunotherapy, peptide vaccine is a promising treatment to target virus-associated malignancy because these types of tumor express non-self viral antigens that are not ignored by immune cells (107). EBV-related proteins are ideal antigens for the peptide vaccine in NNKTL treatment. Kobayashi et al. (108) previously found an epitope peptide, which could bind to promiscuous MHC Class II (HLA-DR9, HLA-DR53, or HLA-DR15), by computer-based peptide algorithm from EBV LMP1. This peptide was naturally processed and expressed on NNKTL cells and could elicit peptide-specific helper T cells, which displayed Th1 phenotype and cytotoxic activity against NNKTL cell. Because this LMP1 epitope peptide overlaps with an HLA-A2–restricted CD8 T cell epitope, this peptide might have the ability to simultaneously induce antitumor CD4 and CD8 T cells against NNKTL cells.

HGF and its receptor c-Met play an essential role in cell proliferation and are involved in various malignancies. Kumai et al. (109) found that both HGF and c-Met were expressed in NNKTL cells and NNKTL tissues, and this pathway activated the proliferation of NNKTL cells in an autocrine manner. c-Met was also responsible for TGF-b production, a negative regulator of immune cells, from NNKTL cells. Kumai et al. (109) further found that several c-Met-derived helper T cell epitope peptides, which are restricted by various HLA-DR molecules. These peptides could elicit c-Met-reactive CD4 T cells that have a cytolytic ability to NNKTL and several solid tumor cells.

Taken together, LMP-1 and c-Met are promising antigens for a peptide vaccine to treat NNKTK patients, and c-Met blockade may augment the antitumor function of peptide-reactive T cells by suppressing TGF-b production from NNKTL cells.

### Future Prospective

The pathogenesis and molecular biology of NNKTL have been gradually revealed as mentioned above. Some of these findings have been considered as direct evidence to establish current promising treatment in NNKTL (81, 105, 106). Although prospective clinical trials are required, novel chemotherapeutic approaches such as MPVIC-P and SMILE have shown favorable clinical outcomes (92, 104). Despite these successes, further basic and translational researches are required to improve the prognosis of NNKTL patients. The blockade of cytokine or chemokine (IL-9, IL-10, CCL17, or CCL21) to inhibit NNKTL proliferation can be an attractive method to treat NNKTL. Recently, several antibodies against cytokine have been approved in the clinic. Mogamulizumab, a clinical-grade anti-CCR4 antibody is a promising candidate to treat NNKTL as shown by Kumai et al. (70). It is mandatory to test the safety of these novel agents in an *in vivo* model. The development of immune deficient mice, in which we succeeded to engraft NNKTL cells, enable us to investigate the effect and safety of these agents (81, 110).

The ligands to pattern-recognition receptors have been recognized as efficient adjuvants in peptide vaccines (111). EBV LMP-1- or c-Met-derived peptide can be a useful peptide vaccine to treat NNKTL patients when combined with adjuvants such as poly-IC or gardiquimod (109, 111). The combination of c-Met or checkpoint blockade with peptide vaccine would be an attracting treatment option in NNKTL. The potential of immunotherapy against NNKTL has been summarized in a review article (112). Because NNKTL is an EBV-related disease, gene therapy to knockout EBV-related proteins or miR would be a fundamental solution to remove NNKTL cells. Since EBV is a widely disseminated virus, the prophylactic vaccine is difficult to establish. However, there is a possibility that high risk EBV subtypes with gene mutation cause EBV-associated malignancies including NNKTL (55–58). Thus, the vaccine that targets high risk EBV can be a preventive vaccine for NNKTL as well as other EBV-associated malignancies.

**Figure 3:**
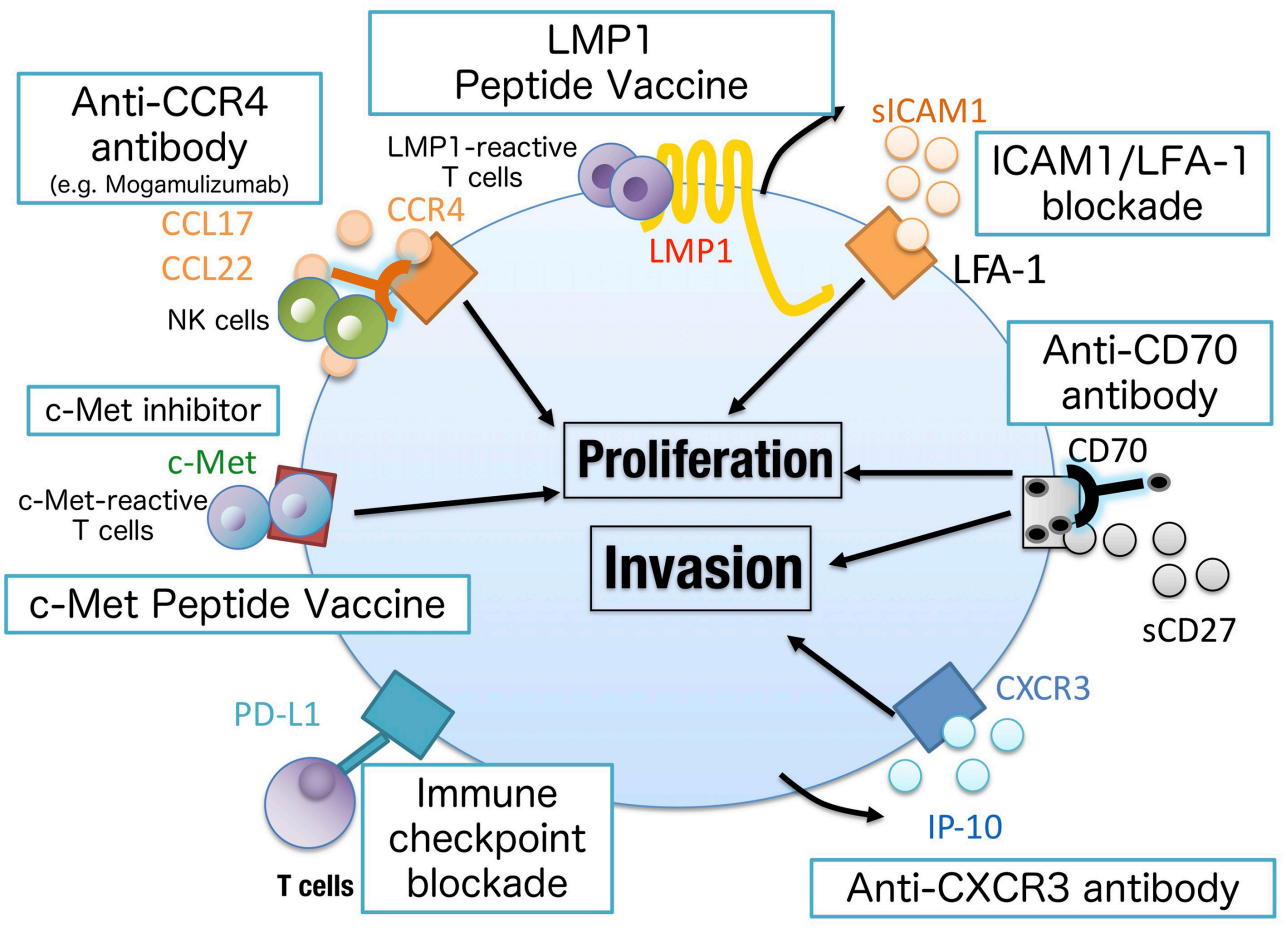
Novel approaches to treat NNKTL. Chemokine/cytokine blockade may inhibit the growth of NNKTL cells (IL-9, IL-10, CXCR3, or LFA-1 blockade) as well as c-Met inhibitor. The antibody against surface markers on NNKTL can directly lyse tumor cells by antibody-dependent cellular cytotoxicity or complement-dependent cytotoxicity. CCR4 or CD70 could be a promising target in this approach. Mogamulizumab, an anti-CCR4 antibody, has been clinically approved to treat cutaneous T cell lymphoma. LMP1 or c-Met peptide vaccine is useful to elicit tumor-specific T cell responses. Because NNKTL cells express PD-L1 to attenuate antitumor T cell responses, immune checkpoint blockades have shown clinical activity in NNKTL patients.

In conclusion, we demonstrated that recent advances in the molecular understanding of NNKTL have led us to establish novel approaches to treat NNKTL patients (Figure 3). We believe that further investigation will make NNKTL a curable disease.

## Author Contributions

All authors listed have made a substantial, direct and intellectual contribution to the work, and approved it for publication.

### Conflict of Interest Statement

The authors declare that the research was conducted in the absence of any commercial or financial relationships that could be construed as a potential conflict of interest.
